# Epigallocatechin-3-Gallate Reduces Hepatic Oxidative Stress and Lowers CYP-Mediated Bioactivation and Toxicity of Acetaminophen in Rats

**DOI:** 10.3390/nu11081862

**Published:** 2019-08-10

**Authors:** Hsien-Tsung Yao, Chien-Chun Li, Chen-Hui Chang

**Affiliations:** 1 Department of Nutrition, China Medical University, 91 Hsueh-shih Road, Taichung 404, Taiwan; 2 Department of Nutrition, Chung Shan Medical University, 110 Sec.1, Jianguo North Road, Taichung 40201, Taiwan

**Keywords:** epigallocatechin-3-gallate, acetaminophen, cytochrome P-450, bioactivation, apoptosis, autophagy, hepatotoxicity

## Abstract

Epigallocatechin-3-gallate (EGCG) is the most abundant polyphenol in green tea. To investigate the effects of dietary EGCG on oxidative stress and the metabolism and toxicity of acetaminophen in the liver, rats were fed diets with (0.54%) or without EGCG supplementation for four weeks and were then injected intraperitoneally with acetaminophen (1 g/kg). The results showed that EGCG lowered hepatic oxidative stress and cytochrome P450 (CYP) 1A2, 2E1, and 3A, and UDP-glucurosyltransferase activities prior to acetaminophen injection. After acetaminophen challenge, the elevations in plasma alanine aminotransferase activity and histological changes in the liver were ameliorated by EGCG treatment. EGCG reduced acetaminophen-induced apoptosis by lowering the Bax/Bcl2 ratio in the liver. EGCG mildly increased autophagy by increasing the LC3B II/I ratio. Lower hepatic acetaminophen–glutathione and acetaminophen–protein adducts contents were observed after EGCG treatment. EGCG increased glutathione peroxidase and NAD(P)H quinone 1 oxidoreductase activities and reduced organic anion-transporting polypeptides 1a1 expression in the liver after acetaminophen treatment. Our results indicate that EGCG may reduce oxidative stress and lower the metabolism and toxicity of acetaminophen. The reductions in CYP-mediated acetaminophen bioactivation and uptake transporter, as well as enhanced antioxidant enzyme activity, may limit the accumulation of toxic products in the liver and thus lower hepatotoxicity.

## 1. Introduction

Studies have shown that intake of green tea or green tea polyphenols (GTPs) can reduce the development and progression of various diseases such as cancer, cardiovascular disease, and neurodegenerative diseases [1,2]. The principal hypothesis associated with the benefits of green tea is related to the strong free radical scavenging, antioxidant, and anti-inflammatory properties of these polyphenol compounds [3,4,5]. In addition, GTPs can change drug metabolism by modulating drug-metabolizing enzymes and transporters [6]. These actions may change the fate of drug metabolism and toxicity. Because of the many polyphenolic components in GTPs, discrepancies exist concerning their effects on drug metabolism and toxicity [6].

Among the various tea polyphenols, epigallocatechin-3-gallate (EGCG) is the most abundant and active polyphenol in green tea. Recently, research into the beneficial effects of green tea on health promotion has focused on EGCG [2]. Studies have shown that EGCG can protect the liver from thioacetamide and triptolide-induced hepatotoxicity [7,8]. However, some studies have also shown that high-dose EGCG administration to animals (through an intragastric tube or intraperitoneal injection) can cause oxidative damage to the liver [9,10,11]. To our knowledge, the dose and route by which EGCG is given to animals can be important factors in determining whether oxidative damage will occur. To date, various commercial EGCG products are on the market worldwide. However, little is known about the effect of dietary EGCG on oxidative stress and drug-metabolizing systems, especially its effect on the metabolism and toxicity of prescribed drugs such as acetaminophen (*N*-acetyl-*p*-aminophenol, APAP). Therefore, it is of considerable importance to evaluate the interactions between EGCG and APAP and their effects on hepatotoxicity.

APAP is widely used as an over-the-counter analgesic and antipyretic agent. APAP overdose is now the most common cause of acute hepatic failure [12]. APAP is metabolized primarily by glucuronidation and sulfation reactions to produce the nontoxic metabolites APAP–glucuronate and APAP–sulfate [13]. APAP overdose can increase the cytochrome P-450 (CYP)-mediated bioactivation of APAP to form a highly reactive metabolite, *N*-acetyl-*p*-benzoquinone imine (NAPQI), which exerts its toxicity by covalent binding to cellular macromolecules [14]. In addition, NAPQI reacts with glutathione (GSH), leading to cellular GSH exhaustion, mitochondrial damage, and cell apoptosis in the liver [15,16]. The removal of damaged organelles, including mitochondria, by autophagy can protect hepatocytes against APAP-induced mitochondrial damage and subsequent necrosis [17]. The other way to lower APAP toxicity is to facilitate the excretion of glucuronate, sulfate, GSH conjugates, and oxidative stress products from the liver by increasing the expression of membrane transporters such as multidrug resistance-associated protein (Mrp)2/3 or reduced uptake transporters such as organic anion-transporting polypeptide (OATP) 1a1 and OATP 1b2 [18,19,20].

Administration of GTPs has been shown to provide protection against APAP-induced liver injury [21]. In our pilot study, supplementation with EGCG (0.54%, *w*/*w*) in the diet for one week had an inhibitory effect against APAP-induced liver injury in rats [22]. However, the reactive oxygen species (ROS) level in the liver might also have been increased by EGCG treatment (Table S1), suggesting that the oxidative stress was mildly increased by short-term exposure of EGCG. In addition, the mechanism by which EGCG lowers APAP-induced liver damage is still not clear. In the present study, rats were fed a diet containing EGCG for a longer time (four weeks) to investigate the effects of EGCG on oxidative stress, drug-metabolizing enzymes, and membrane transporters in the liver. Then, the effects of EGCG on the metabolism and toxicity of APAP in the liver were investigated.

## 2. Materials and Methods

### 2.1. Materials

Methoxyresorufin, *p*-nitrophenol, 4-nitrocatechol, testosterone, resorufin, GSH, NADPH, 1-chloro-2,4-dinitrobenzene, and heparin were obtained from Sigma (St. Louis, MO, USA). 6-β-Hydroxytestosterone was purchased from Ultrafine Chemicals (Manchester, UK). EGCG (purity > 99%) was purchased from Huzhou Ruzhou Rongkai Foliage Extract Co. LTD (Huzhou, China). All other chemicals and reagents were of analytical grade and were obtained commercially.

### 2.2. Animal Studies

Experiment I. Male Sprague–Dawley (SD) rats (six weeks old), obtained from BioLASCO in Ilan, Taiwan, were used to investigate the effect of dietary EGCG on oxidative stress and the activities of drug-metabolizing enzymes and the expressions of membrane transporters in the liver. Rats were fed a standard laboratory chow powder diet (Purina Laboratory Chow 5001) without (control group) or with 0.18% EGCG (1× EGCG group) or 0.54% EGCG (3× EGCG group) for four weeks. Each group consisted of five rats. Rats were fed a laboratory diet containing 0.54% EGCG, which was approximately equivalent to the dose (daily dose: 460 mg/kg body weight) used in previous studies that found that EGCG did not cause liver damage [22,23]. Rats were housed in plastic cages in a room kept under a 12-h light–dark cycle and 23 ± 1 °C with 60% ± 5% relative humidity. Food and drinking water were available ad libitum for four weeks.

At the end of the experiment, fresh feces was collected for determining microbial β-glucuronidase activity. Then, rats were sacrificed under carbon dioxide (70:30, CO_2_/O_2_) anesthesia, and blood was collected by exsanguination via the abdominal aorta. Plasma was separated from the blood by centrifugation (1750× *g*) at 4 °C for 20 min using heparin as the anticoagulant agent. Plasma alanine aminotransferase (ALT) activity was measured immediately by use of commercial kit (Randox Laboratories, Antrum, Loughborough, UK). The liver samples from each animal were stored at −80 °C.

Experiment II. Male SD rats (six weeks old) were randomly divided into three groups with six rats in each group to investigate the effects of EGCG on the metabolism and toxicity of APAP. The animals in the control and APAP groups were fed a standard laboratory chow powder diet. The animals in APAP + EGCG were fed the same diet fortified with 0.6% EGCG (daily dose: 511 mg/kg body weight). At the end of the four-week feeding period, food was withdrawn for 12 h. A single 1000 mg/kg (BW) dose of APAP, as a solution in polyethylene glycol 400/water (50/50, *v*/*v*), was intraperitoneally injected into each animal in the APAP and APAP + EGCG groups. The control animals were injected with the above vehicle only. At 12 h after the APAP dose, the animals were sacrificed. The separated plasma, liver, and urine samples (collected in metabolic cage) were used to determine APAP and its conjugates. Part of the liver samples was excised and fixed in 10% neutral formalin followed by dehydration in ascending grades of alcohol, clearing in xylene, and embedding in paraffin wax. A liver section 5 μm thick was stained with hematoxylin and eosin (H&E) for histological examination [24].

This study was approved (No. 102-70-N) by the Animal Center Management Committee of China Medical University. The animals were maintained in accordance with the guidelines for the care and use of laboratory animals [25].

### 2.3. Drug-Metabolizing Enzyme Activity Assays

Two-step centrifugation was used to prepare liver microsomes and cytosol preparations according to a method reported previously [26]. The formation of metabolites from various CYP enzyme reactions was determined by high-performance liquid chromatography (HPLC)/mass spectrometry (MS) [26]. Testosterone (60 μM), midazolam (2.5 μM), methoxyresorufin (5 μM), *p*-nitrophenol (50 μM), and were used as the probe substrates for testosterone 6β-hydroxylation (CYP3A), midazolam 1-hydroxylation (CYP3A), methoxyresorufin *O*-demethylation (CYP1A2), and *p*-nitrophenol 6-hydroxylation (CYP2E1), respectively. Enzyme activities were expressed as pmol of metabolite formation/min/mg protein.

Microsomal UDP-glucuronosyltransferase (UGT) activity was determined by using *p*-nitrophenol as the substrate and the formation of *p*-nitrophenol glucuronic acid was measured by HPLC/MS [27]. Cytosolic sulfotransferase activity was determined by using phosphoadenosine 5-phosphosulphate as the substrate and *p*-nitrophenol as the acceptor of sulfate, and the formation of of adenosine 3,5-diphosphate was measured by HPLC/MS [28]. The activities of glutathione *S*-transferase (GST) [29] and NAD(P)H quinone 1 oxidoreductase (NQO1) [30] were determined spectrophotometrically.

### 2.4. Determination of Oxidative Stress in the Liver

Liver homogenate was prepared by homogenizing 1 g of liver with 10 mL of 1.15% KCl and centrifuging the homogenate at 10,000 × *g* for 15 min at 4 °C. The resulting supernatant was used to determine the oxidative markers including GSH, lipid peroxide, ROS, and glutathione peroxidase activity. The GSH content in liver homogenates was determined by HPLC/MS [31]. GSH peroxidase activity was determined spectrophotometrically according to the method of Mohandas et al. [32]. Liver thiobarbituric acid–reactive substance (TBARS) content was determined by the method of Uehiyama and Mihara [33]. ROS production was measured according to the method of Ali et al. [34] by determining the fluorescent product of dichlorofluorescein.

### 2.5. APAP and APAP Conjugates in the Plasma, Liver, and Urine

Plasma, urine, and liver homogenate samples were diluted and extracted by use of acetonitrile and were then analyzed by HPLC/MS [35]. To remove low molecular weight compounds with the potential to interfere in the assay, liver homogenate was filtered through a Nanosep centrifugal device (Pall Life Sciences, Ann Arbor, MI, USA) with a membrane molecular weight cutoff of 30 kDa to determine hepatic APAP–protein adducts. The filtrate was then digested for 16 h with proteases to free the APAP–cysteine from APAP–protein adducts [35]. The resulting APAP–cysteine level was determined by HPLC/MS [36].

### 2.6. In Vitro APAP–GSH Formation

To evaluate the susceptibility of APAP–GSH formation in rat liver microsomes from each group, amounts of 20 mM APAP and 5 mM GSH were incubated with liver microsomes (1 mg/mL protein) containing 2 mM NADPH, 1.5 mM MgCl_2_, and 45 mM potassium phosphate buffer (pH 7.4). The reaction was incubated at 37 °C for 60 min. An electrophilic metabolite, NAPQI, conjugated with GSH to form APAP–GSH during the reaction. The formation of APAP–GSH in the incubation was then added with an equal volume of ice-cold isopropanol to stop the reaction. The APAP–GSH formation was determined by HPLC/MS [37].

### 2.7. Immunoblotting Analysis

Liver homogenate was prepared by homogenizing 1 g of liver with 10 mL of 1.15% KCl and centrifuging the homogenate at 10,000× *g* for 15 min at 4 °C. The resulting supernatant was used to determine the protein expressions of BCL2-associated X protein (Bax), B-cell lymphoma 2 (Bcl2), and microtubule-associated protein light chain 3B (LC3B I/II). The supernatant was used as a cellular protein for sodium dodecyl sulfate polyacrylamide gel electrophoresis (SDS-PAGE) and Western blot analysis [26]. Plasma membrane was prepared by use of a cell membrane protein extraction kit (Bio-Kit, Miaoli, Taiwan). After electrophoresis, the separated proteins were transferred to polyvinylidene fluoride membranes (Millipore, Billerica, MA, USA). The membrane was then blocked with 5% nonfat milk, followed by probing with primary antibodies against CYP1A2, CYP2E1, CYP3A, Bax, Bcl2, LC3B I/II, Mrp2/3, p-glycoprotein (p-gp), and Oatp1a1 [38]. The membranes were then probed with the horseradish peroxidase-labeled secondary antibody. The bands of target proteins were visualized and quantified as described by Yen et al. [38].

### 2.8. Fecal β-Glucuronidase Activity

Fecal β-glucuronidase activity was determined by the method of Yao and Chiang [39]. Nitrophenyl-β-d-glucuronide was used as the substrate. Fecal β-glucuronidase activity was expressed as nmol *p*-nitrophenol formation/min/mg protein.

### 2.9. Statistical Analysis

One-way ANOVA (SAS Institute, Cary, NC, USA) was used to calculate statistical differences among groups. The differences were considered to be significant at *p* < 0.05 as determined by independent-sample *t*-tests.

## 3. Results

### 3.1. Drug-Metabolizing Enzyme Activity, Oxidative Stress, Membrane Transporters, and Liver Function Index in Normal Rats

In experiment I, we evaluated the effects of supplementation with EGCG for four weeks on oxidative stress, drug-metabolizing enzyme activities and membrane transporters in the liver of normal rats. Table 1 shows the effects of EGCG on drug-metabolizing enzyme activity and oxidative stress in the liver. Rats fed the 3× (0.54%) EGCG diet showed significantly lowered (*p* < 0.05) activity of CYP3A, CYP2E1, and CYP1A2 in the liver compared to rats fed the control diet. UGT and GST activities were also reduced in the 3× EGCG group (*p* < 0.05). In addition, the GSSG content was lower and the GSH/GSSG ratio was higher in the livers of rats fed the 1× or 3× EGCG diet than in animals fed the control diet (*p* < 0.05). Hepatic TBARS and ROS levels were also lower after 3× EGCG treatment (*p* < 0.05). These results indicated that EGCG may reduce drug metabolism and oxidative stress in the liver. No significant difference in plasma ALT activity was observed among the groups, indicating that rats fed the 3× EGCG diet for four weeks caused no hepatotoxicity.

**Figure 1:**
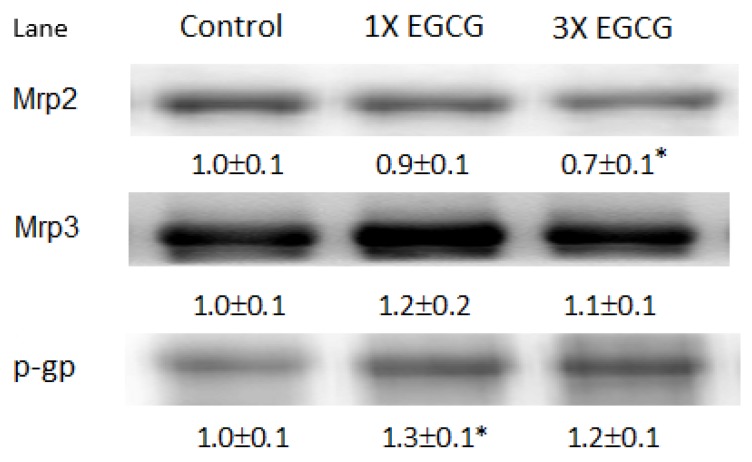
Effects of EGCG on membrane transporters in the livers of rats. Densitometry quantitation of membrane transporter protein (Mrp2/3 and *p*-glycoprotein, p-gp) levels from rats fed the EGCG diet for four weeks. Each lane represents the pooled liver membrane protein from five to six individual rats per group. The protein band was quantified by densitometry, and the level of the control was set at 1. EGCG, epigallocatechin-3-gallate. * Significantly different from control group, *p* < 0.05.

Figure 1 shows the immunoblots of Mrp2/3 and *p*-glycoprotein in the liver of control and EGCG-treated rats. Mrp2 protein expression was mildly lower in rats fed the 3× EGCG diet than in the control group (*p* < 0.05). EGCG had no significant effect on the expression of Mrp3 protein in the rat liver (*p* > 0.05). Rats fed the 1× EGCG diet caused a small increase in *p*-glycoprotein expression (*p* < 0.05).

On the other hand, in this study, EGCG supplementation reduced (*p* < 0.05) fecal microbial β-glucuronidase activity (control group: 79.6 ± 32.1 nmol/min/mg protein; 3× EGCG group: 31.3 ± 16.3 nmol/min/mg protein). There were no significant differences (*p* > 0.05) in food intake, body weight, or liver weight between rats fed the EGCG-containing diet and animals fed the control diet.

**Figure 2:**
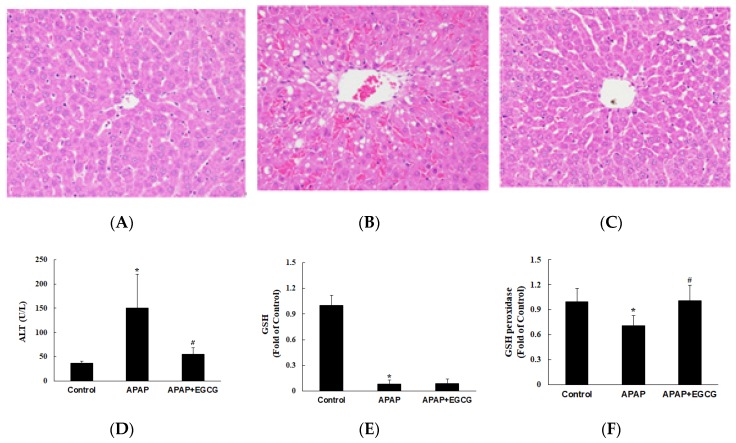
Effects of EGCG supplementation (0.6%) on APAP-induced hepatotoxicity in rats. Histopathological examination of livers was shown in (**A**) the control group, (**B**) the APAP group, and (**C**) the APAP + EGCG group. H&E stain, 400×. Normal architecture of the liver was found in the control group (**A**). Multifocal necrosis was graded as slight (2) in (**B**) (APAP group) and minimal (1) in (**C**) (APAP + EGCG group). Plasma alanine aminotransferase (ALT) activity and hepatic reduced- glutathione (GSH) and GSH peroxidase activity were shown in (**D**–**F**), respectively. * Significantly different from control group, *p* < 0.05. # Significantly different from APAP group, *p* < 0.05.

In experiment II, the effect of EGCG on the metabolism and toxicity of APAP was evaluated. Rats were fed a normal diet or a normal diet containing EGCG (0.6%) for four weeks. To confirm the pattern of hepatotoxicity and compare the extent of liver damage between the control and the EGCG-fed animals (Figure 2A–C), histological examination of H&E stained liver sections was conducted 12 h after APAP challenge. Plasma ALT activity was significantly increased 12 h after APAP treatment compared with that in control animals (*p* < 0.05) (Figure 2D). However, plasma ALT activity was significantly lower (*p* < 0.05) in rats treated with EGCG after APAP treatment. It was worth noting that the plasma transaminase observations were consistent with morphological findings. Histopathological changes in the liver came with significant necrosis and degeneration of hepatocytes in the centrilobular region and with perivenular inflammatory infiltrates after APAP treatment (Figure 2B). These results indicate that APAP-induced histopathological changes and hepatotoxicity were significantly ameliorated by EGCG treatment (Figure 2C).

To investigate the effects of EGCG supplementation on oxidative stress in the liver, the hepatic GSH, GSH peroxidase, and TBARS were determined. As shown in Figure 2E,F, the GSH level in the liver was dramatically decreased (*p* < 0.05) and GSH peroxidase activity was lower (*p* < 0.05) in the APAP group than in the control group. EGCG had no significant effect on hepatic GSH content after APAP treatment (*p* > 0.05). However, EGCG increased (*p* < 0.05) GSH peroxidase activity after APAP treatment. There was no significant difference on hepatic TBARS content (nmol/g protein) among the groups (control group: 179.9 ± 58.9; APAP group: 205.6 ± 75.9; APAP + EGCG group: 221.5 ± 72.2). In this study, there were no significant differences (*p* > 0.05) in food intake, liver weight, or body weight in the APAP group compared with the untreated control group.

### 3.2. Apoptosis and Autophagy in the Liver

**Figure 3:**
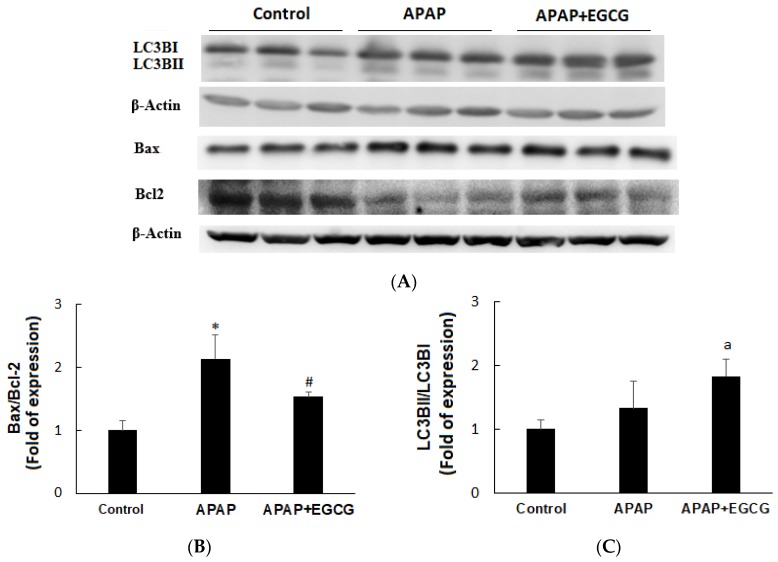
Effects of EGCG on APAP-induced apoptosis (Bax/Bcl2) and autophagy (LC3B II/ILC3B I) in the rat liver (**A**). β-actin served as the loading control. Values are given as the mean ± S.D. (*n* = 3). Densitometric analysis for Bax/Bcl2 (**B**) and LC3B II/ILC3B I (**C**) protein levels is shown. APAP, N-acetyl-p-aminophenol. * Significantly different from control group, *p* < 0.05. ^#^ Significantly different from APAP group, *p* < 0.05. ^a^ Significantly different from APAP group, *p* < 0.1.

Apoptosis and autophagy indices in the liver are shown in Figure 3A. Rats treated with APAP induced apoptosis by increasing Bax protein expression and decreasing Bcl2 protein expression compared with the vehicle-treated control animals (*p* < 0.05). A mild increase (*p* > 0.05) in LC3BI and LC3BII protein was found in the APAP group. EGCG had no effect on Bax expression, but significantly increased (*p* < 0.05) Bcl2 expression after APAP treatment, resulting in a higher Bax/Bcl2 ratio (*p* < 0.05) (Figure 3B). The hepatic LC3BII/LC3BI ratio was not affected (*p* > 0.05) by APAP (Figure 3C). However, EGCG mildly increased hepatic LC3BII/LC3BI ratio (*p* < 0.1) after APAP treatment.

### 3.3. APAP and APAP Conjugates in the Plasma, Liver, and Urine

At 4 h after intraperitoneal injection of a single dose of APAP, the APAP–glucuronide concentration in the plasma was reduced (*p* < 0.05) by EGCG treatment (Table 2). APAP, APAP–sulfate, and APAP–GSH concentrations in the plasma were not changed by EGCG treatment (*p* > 0.05). The APAP–glucuronide content in the liver tended to be lowered by EGCG treatment, but this change did not reach statistical significance (*p* > 0.05). However, APAP–protein adducts were significantly lowered (*p* < 0.05) by EGCG treatment. APAP, APAP–sulfate, and APAP–GSH contents in the liver were not changed (*p* > 0.05) by EGCG treatment. These results suggested that, in the first 4 h after injection of APAP, the lower CYP-medicated APAP bioactivation could be ameliorated by EGCG treatment.

At 12 h after APAP injection, the contents of APAP–GSH and APAP–protein adducts in the liver were significantly lowered by EGCG treatment (*p* < 0.05). Plasma concentrations of APAP, APAP–sulfate, and APAP–glucuronide, however, did not differ significantly with respect to EGCG supplementation. Notably, the urinary excretion of APAP was slightly but significantly higher (*p* < 0.05) in EGCG-supplemented rats (APAP group: 3.3 mg/12 h; APAP + EGCG group: 5.4 mg/12 h). However, this mild increase in the APAP level in urine due to EGCG may not explain the hepatoprotective effect of EGCG. No significant differences (*p* > 0.05) in APAP–sulfate or APAP–glucuronide content in urine were noted. There was no significant difference (*p* > 0.05) in urine volume after APAP challenge for 12 h (APAP group: 12.5 ± 4.3 mL; APAP + EGCG group: 14.1 ± 1.3 mL).

The in vitro formation of APAP–GSH in liver microsomes of APAP-treated rats was evaluated. Similar to the results of a previous report [37], the results showed that APAP treatment for 12 h increased (*p* < 0.05) the susceptibility of APAP–GSH formation in rat liver microsomes. However, in this study, EGCG supplementation for four weeks had no significant effect (*p* > 0.05) on the in vitro APAP–GSH formation rate (pmol/min/mg protein) (control group: 139.6 ± 34.0; APAP group: 268.9 ± 60.0; APAP + EGCG group: 229.2 ± 35.4).

### 3.4. Drug-Metabolizing Enzyme Activity in APAP-Treated Rats

The hepatic drug-metabolizing enzyme activity was reduced after APAP treatment (Table 3). Midazolam 1-hydroxylase (CYP3A), nitrophenol 6-hydroxylase (CYP2E1), UGT, NQO1, and GST activities in the liver were lower (*p* < 0.05) after APAP challenge. EGCG had no significant effects on these enzyme activities (*p* > 0.05). No difference in methoxyresorufin *O*-demethylase (CYP1A2) activity was observed between the groups (*p* > 0.05). Sulfotransferase activity was not affected by APAP, but EGCG supplementation reduced sulfotransferase activity after APAP treatment (*p* < 0.05). Notably, EGCG increased NQO1 activity after APAP treatment (*p* < 0.05).

### 3.5. Membrane Transporters’ Expression

**Figure 4:**
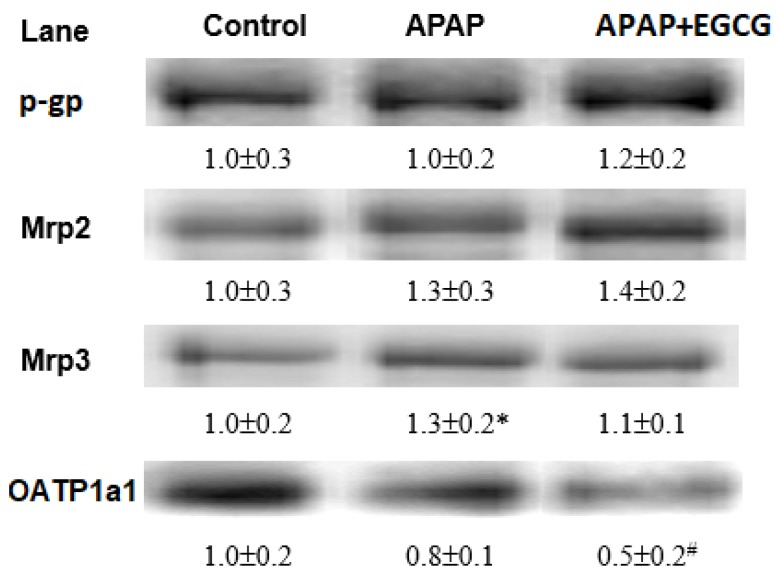
Densitometry quantitation of Mrp2/3, p-gp, and OATP1a1 protein expression from rats fed an EGCG diet for four weeks and then treated with APAP for 12 h. Each lane represents the pooled (*n* = 5–6) liver membrane protein from an individual rat. The protein band was quantified by densitometry, and the level of the control was set to 1. * Significantly different from control group, *p* < 0.05. ^#^ Significantly different from APAP group, *p* < 0.05.

Immunoblots of liver membrane transporters are shown in Figure 4. Mrp2 and Mrp3 expression was mildly increased and that of OATP 1a1 was mildly decreased by 12 h of APAP treatment (*p* > 0.05). EGCG had no significant effect on the protein expressions of p-gp, Mrp2, or Mrp3 (*p* > 0.05). However, EGCG significantly reduced (*p* < 0.05) the OATP 1a1 expression in the liver.

## 4. Discussion

In the present study, the results showed that EGCG supplementation for four weeks significantly reduced oxidative stress and the activities of several drug-metabolizing enzymes in the rat liver. After challenge with APAP, EGCG reduced CYP-mediated APAP bioactivation and apoptosis and mildly increased autophagy in the liver. In addition, EGCG increased the activities of antioxidant enzymes, including GSH peroxidase and NQO-1, and decreased the expression of the uptake membrane transporter, OATP 1a1, after APAP treatment. These results indicate that dietary EGCG may reduce APAP-induced hepatotoxicity by lowering CYP-mediated APAP bioactivation, increasing antioxidant enzyme activity, and reducing the accumulation of toxic products in the liver.

EGCG shows both antioxidant and pro-oxidant effects in biological systems. EGCG acts as a pro-oxidant compound when it undergoes metabolic processes that produce ROS [9]. At high doses, the oxidized form of EGCG is EGCG o-quinone, which reacts with glutathione to form thiol conjugates, resulting in the accumulation of EGCG o-quinone in hepatocytes and causing liver damage [40]. In our preliminary study, EGCG supplementation in the diet (0.54%, *w*/*w*) caused a transient increase in ROS level in the liver during the first week of EGCG treatment, but the production of ROS may act as a signal to upregulate GSH synthesis and GSH peroxidase activity in the liver. Therefore, EGCG did not cause any hepatotoxicity (Table S1). In this study, hepatic ROS, GSSG, and TBARS contents were significantly decreased after four weeks of treatment with the same dose of EGCG, indicating that the oxidative stress in the liver was diminished. This phenomenon could be partly explained by the rats having adapted to the high dose of EGCG (0.6% in the diet) and maintained a high antioxidant capacity in the liver after four weeks of EGCG treatment. The other possibility is that EGCG supplementation in the diet may have lowered the intestinal absorption rate of EGCG compared with EGCG administered to the animal by intragastric [10] or intraperitoneal injection [11], resulting in a lower concentration of plasma EGCG. Therefore, in this study, EGCG administration for four weeks reduced oxidative stress in the liver and caused no hepatotoxicity.

After 12 h of administration of a single dose of APAP to rats, APAP caused liver damage, which was characterized by an increase in the plasma ALT concentration, a change in hepatocyte morphology, a dramatic decrease in GSH content, and a decrease in liver GSH peroxidase activity (Figure 2). In addition, APAP increased the Bax/Bcl2 ratio with little or no change in the LC3B-II/LC3B-I ratio in the liver, indicating that APAP induced hepatocyte apoptosis without affecting autophagy. Notably, treatment with EGCG caused a lower Bax/Bcl2 ratio and a higher LC3B II/LC3B I ratio, indicating that EGCG could reduce apoptosis and induce autophagy (Figure 3B,C). Activation of autophagy by EGCG has been demonstrated to protect against APAP-induced hepatotoxicity [41]. These results suggest that inhibition of apoptosis and induction of autophagy after EGCG challenge may protect the liver against APAP-induced hepatotoxicity.

In this study, the increased GSH peroxidase activity due to EGCG may have reduced ROS production during CYP-mediated APAP metabolism. EGCG has been shown to be proficient at scavenging free radicals [5]. Therefore, the reduced oxidative stress by EGCG after APAP challenge may be partially attributed to its direct and/or indirect increase in antioxidant activity or decrease in ROS production in the liver, even though the oral bioavailability of EGCG is low (<1%) [42].

Regarding the drug-metabolizing enzyme activity, a previous study showed that CYP3A, sulfotransferase, and GST enzyme activities in the liver were significantly reduced after one week of EGCG feeding (0.54%, *w*/*w*) [22]. In this study, hepatic CYP3A, CYP2E1, CYP1A2, UGT, and GST activity was suppressed after four weeks of EGCG feeding. This observation is consistent with previous results showing that orally administered EGCG reduces the activities of hepatic drug-metabolizing enzymes [6,22,43]. These results suggest that dietary EGCG may lower the metabolism of drugs or toxic compounds in the liver.

Consistent with previous findings, in this study, APAP treatment for 12 h reduced the activity of drug-metabolizing enzymes and antioxidant enzymes. It is known that APAP-induced liver toxicity is mediated by covalent binding to critical proteins or enzymes with NAPQI [44]. A toxic dose of APAP to animals decreases the catalytic activity of the hepatic enzymes, including CYP enzymes, UGT, GST, and glutathione peroxidase, probably due to covalent binding to these cellular proteins [22,45]. In addition to CYP2E1 and CYP1A2, CYP3A is an important enzyme responsible for the CYP-mediated bioactivation of APAP to generate the electrophile NAPQI in both human and rats, especially when an overdose of APAP is administered [35,46,47,48]. In this study, EGCG administration caused lower hepatic CYP3A, CYP2E1, and CYP1A2 activity prior to APAP injection (Table 1). These changes caused by EGCG may result in a lower CYP-mediated NAPQI production after APAP treatment. Indeed, a lower formation of APAP–GSH and APAP–protein adducts was observed in the liver (Table 3). In addition to APAP–protein adducts, APAP–GSH is toxic to the liver because the conjugate can induce mitochondrial impairment, which can lead to enhanced ROS production [49]. In this study, EGCG increased NQO1 activity after APAP treatment, which may enhance the conversion of NAPQI back to the parent APAP. Therefore, EGCG suppressed CYP enzyme activity prior to APAP injection and had higher NQO1 activity after APAP challenge resulted in lower NAPQI production, which might lead to lower formation of APAP–protein adducts and APAP–GSH in the liver (Table 3). Therefore, EGCG lowering APAP-induced hepatotoxicity is likely due to its ability to reduce CYP enzyme activity and enhance NQO1 activity and, thus, lower CYP-mediated APAP bioactivation.

Several studies have indicated that the expression of efflux membrane transporters such as Mrp2/3 and p-glycoprotein is increased after APAP intoxication [18,19,50]. In general, APAP–glucuronide and APAP–GSH are mainly excreted into bile, while APAP–sulfate is mainly excreted into urine [51]. These membrane proteins can remove toxic metabolites and oxidative products from the liver via the urine or bile. Uptake transporters of OATPs in the liver, such as Oatp1a1 and Oatp1b2, which mediate the uptake of numerous drugs and xenobiotics into cells, were reduced after APAP treatment [18]. Roth et al. [52] showed that the expression of Oatp1a1 is inhibited by EGCG. In this study, the protein expression of Mrp2 and Mrp3 was slightly increased and that of OATP1a1 was decreased after 12 h of APAP treatment. EGCG had no effect on Mrp2/3 and p-glycoprotein protein expressions in the liver; however, EGCG significantly reduced (−37.5%) OATP1a1 expression. These results suggest that EGCG may reduce the hepatic uptake of APAP and its metabolites from the circulation into the liver. On the other hand, in this study, EGCG supplementation also lowered fecal β-glucuronidase activity, which might diminish deconjugation of APAP–glucuronide and thus increase fecal APAP–glucuronide excretion. This observation is similar to the results of a previous study [53]. Therefore, the reduced hepatic OATP1a1 protein expression and microbial β-glucuronidase activity due to EGCG may lead to lower reabsorption of APAP into the liver from the circulation and intestine, respectively. These actions may lower repeated CYP-mediated APAP bioactivation in the liver.

In summary, the results of this study show that EGCG supplementation for four weeks reduced APAP-induced liver damage in rats. The mechanisms contributing to the detoxification of APAP by EGCG may include reduced CYP-mediated APAP bioactivation, oxidative stress, and apoptosis; and increased autophagy and lower accumulation of toxic products in the liver.

## Figures and Tables

**Table 1:** Drug-metabolizing enzyme activities, oxidative stress, and liver function index in the liver of rats fed EGCG-containing diets for four weeks ^a^.

	Control	1× EGCG	3× EGCG
**Phase I Enzymes (pmol/min/mg protein)**			
Testosterone 6β-hydroxylase (CYP3A)	525.2 ± 63.0	535.7 ± 44.2	281.1 ± 75.3 *
Nitrophenol 6-hydroxylase (CYP2E1)	304.7 ± 31.0	308.3 ± 38.4	208.9 ± 28.2 *
Methoxyresorufin *O*-demethylase (CYP1A2)	34.5 ± 0.3	35.9 ± 5.3	24.5 ± 2.9 *
**Phase II Enzymes (nmol/min/mg protein)**			
UDP-glucurosyltransferase	34.5 ± 0.3	35.9 ± 5.3	24.5 ± 2.9 *
Sulfotransferase	1.3 ± 0.0	1.3 ± 0.0	1.3 ± 0.0
Glutathione S-transferase	209.1 ± 10.0	178.1 ± 9.4 *	158.2 ± 6.4 *
**Oxidative Stress Status**			
GSH (nmol/mg protein)	46.2 ± 0.9	43.8 ± 3.2	42.9 ± 1.7 *
GSSG (nmol/mg protein)	0.7 ± 0.1	0.3 ± 0.0 *	0.3 ± 0.1 *
GSH/GSSG	69.1 ± 9.6	163.8 ± 27.6 *	139.6 ± 38.1 *
GSH peroxidase (nmol/min/mg protein)	83.0 ± 4.1	87.0 ± 16.3	88.1 ± 7.9
TBARS (nmol/g protein)	116.5 ± 18.0	98.3 ± 0.4	75.6 ± 6.9 *
ROS (nmol/mg protein)	0.96 ± 0.11	0.71 ± 0.03 *	0.74 ± 0.10 *
**Liver Function Index**			
Alanine aminotransferase (U/L)	22.4 ± 3.2	23.7 ± 4.0	21.0 ± 4.4

^a^ Results are expressed as the mean ± S.D. of five rats in each group. * Significantly different from control, *p* < 0.05. EGCG, epigallocatechin-3-gallate. 1× EGCG: 0.18% EGCG in the diet; 3× EGCG: 0.54% EGCG in the diet. UDP-glucurosyltransferase: Uridine-diphospho glucurosyltransferase; GSH: reduced-glutathione; GSSG: oxidized-glutathione; TBARS: thiobarbituric acid–reactive substance; ROS: reactive oxygen species.

**Table 2:** APAP and its metabolites in the plasma, liver, and urine after APAP challenge for 12 h ^a^.

	4 h		12 h	
	APAP	APAP + EGCG	APAP	APAP + EGCG
**Plasma**				
APAP (μg/mL)	259.4 ± 130.3	187.9 ± 18.1	303.1 ± 61.0	387.5 ± 62.9
APAP–glucuronide (μg/mL)	101.2 ± 5.2	82.2 ± 6.9 *	59.5 ± 9.3	51.4 ± 10.9
APAP–sulfate (μg/mL)	224.2 ± 102.9	163.8 ± 23.9	29.1 ± 14.1	19.8 ± 8.7
APAP–GSH (μg/mL)	12.1 ± 9.4	11.9 ± 6.1	32.5 ± 10.9	11.0 ± 9.7 *
**Liver**				
APAP (μg/g liver)	115.7 ± 33.1	128.9 ± 55.6	77.3 ± 31.9	95.9 ± 33.0
APAP–glucuronide (μg/g liver)	618.1 ± 211.7	450.2 ± 89.1	432.5 ± 181.3	520.3 ± 157.8
APAP–sulfate (μg/g liver)	52.5 ± 15.7	69.4 ± 16.1	49.3 ± 18.0	44.9 ± 14.9
APAP–GSH (μg/g liver)	438.6 ± 88.2	447.8 ± 140.2	645.2 ± 230.6	344.2 ± 122.2 *
APAP–protein adducts (mg/g liver)	1.8 ± 0.4	1.1 ± 0.2 *	3.5 ± 1.0	2.1 ± 0.4 *
**Urine**				
APAP (mg/12 h)			3.3 ± 0.7	5.4 ± 0.7 *
APAP–glucuronide (mg/12 h)			144.5 ± 61.5	155.7 ± 14.2
APAP–sulfate (mg/12 h)			209.2 ± 39.1	189.6 ± 13.5

^a^ Results are expressed as the mean ± S.D. of four (for 4 h) or six (for 12 h) rats in each group. APAP, N-acetyl-p-aminophenol. * Significantly different from APAP group, *p* < 0.05.

**Table 3:** Drug-metabolizing enzyme activities in the rat liver after APAP challenge for 12 h ^a^.

	Control	APAP	APAP + EGCG
**Phase I Enzymes (pmol/min/mg protein)**			
Midazolam 1-hydroxylation (CYP3A)	216.1 ± 60.3	124.3 ± 26.9 *	153.6 ± 48.5
Nitrophenol 6-hydroxylase (CYP2E1)	517.6 ± 55.1	388.4 ± 74.8 *	436.4 ± 91.3
Methoxyresorufin *O*-demethylase (CYP1A2)	28.0 ± 2.9	28.0 ± 2.8	26.0 ± 1.9
**Phase II Enzymes (nmol/min/mg protein)**			
UDP-glucurosyltransferase	48.0 ± 6.8	28.9 ± 11.3 *	25.9 ± 3.6
Sulfotransferase	0.73 ± 0.16	0.62 ± 0.1	0.44 ± 0.13 ^#^
Glutathione S-transferase	146.5 ± 43.5	103.9 ± 15.4 *	130.1 ± 30.1
NADPH: quinine oxidoreductase-1	462.4 ± 132.8	279.5 ± 91.3 *	383.5 ± 82.4 ^#^

^a^ Results are expressed as the mean ± S.D. of six rats in each group. * Significantly different from control group; ^#^ significantly different from APAP group, *p* < 0.05.

## Supplementary Materials

The following are available online at https://www.mdpi.com/2072-6643/11/8/1862/s1, Table S1: Oxidative stress status and liver function index in the liver of rats fed the EGCG-containing diets for one weeks.
