# Supplementary material for: Epigallocatechin-3-Gallate Reduces Hepatic Oxidative Stress and Lowers CYP-Mediated Bioactivation and Toxicity of Acetaminophen in Rats
Source: Nutrients. 2019 Aug 10;11(8):1862. doi: 10.3390/nu11081862 (PMC6723635; doi:10.3390/nu11081862)
Supplement: Supplementary file 1 [file nutrients-11-01862-s001.pdf]

Table S1. Oxidative stress status and liver function index in the liver of rats fed the EGCG-containing diets for one weeks [a]

Groups	Control	1X EGCG	3X EGCG
GSH (nmol/mg protein)	35.7±7.9	43.4±10.0	55.6±6.1 *
Glutathione peroxidase (nmol/min/mg protein)	51.6±3.0	64.6±12.9	94.0±7.2 *
TBARs (nmol/g protein)	148.1±55.3	172.2±22.3	163.2±43.3
ROS (nmol/mg protein)	0.64±0.07	0.75±0.10	0.90±0.05 *
Aspartate aminotransferase (U/L)	24.3±4.5	25.2±7.4	17.5±1.2
Alanine aminotransferase (U/L)	20.1±2.2	17.9±3.4	17.3±1.0

[a] Results are expressed as the mean±S.D. of five rats in each group. * Significantly different from control, $p<0.05$. 1X EGCG: 018% EGCG in the diet; 3X EGCG: 0.54% EGCG in the diet.
